# Clinical application of the contrast-enhancement boost technique in computed tomography angiography of the portal vein

**DOI:** 10.1007/s00261-022-03754-4

**Published:** 2022-12-05

**Authors:** Juan Hou, Yuan Zhang, Jing Yan, Tieliang Zhang, Wenwen Xia, Yucai Zhu, Wenya Liu

**Affiliations:** 1 Imaging Center, First Affiliated Hospital of Xinjiang Medical University, Urumqi, 830011 Xinjiang China; 2 Imaging Center, The Fourth Affiliated Hospital of Xinjiang Medical University, Urumqi, 830011 Xinjiang China; 3 Canon Medical Systems (China), Co., Ltd., Room 1004-1006, City Point, NO 666 West Huaihai Rd, Changning District, 200052 Shanghai China

**Keywords:** Portal vein, Angiography, Computed tomography, Contrast enhancement boost, Image quality

## Abstract

**Purpose:**

The aim of this study was to explore the improved image quality of the portal vein using the contrast-enhancement boost (CE-boost) technique for the improved visibility of abdominal-enhanced computed tomography (CT) scans in clinical practice.

**Methods:**

This retrospective study included 50 patients in Group A who underwent routine abdominal-enhanced CT and 50 patients in Group B who underwent abdominal computed tomography angiography (CTA) with matched body mass index, age, and sex. Images in Group A were postprocessed with the CE-boost technique for further enhanced visibility of the portal vein. Both subjective and objective assessments of different branches of the portal vein in three types of images (i.e., Group A with CE-boost and without CE-boost, Group B) were statistically analyzed.

**Results:**

The subjective scores of two experienced radiologists showed good consistency (kappa value > 0.624, *p* < 0.001), and the score of Group A with CE-boost (mean, 4.64) was significantly higher than that of the others (*p* < 0.001). The liver parenchyma and most target veins in Group A with CE-boost showed the highest CT, signal-to-noise ratio (SNR), and contrast-to-noise ratio (CNR) values and the lowest standard deviation (SD), while the CNR of most portal veins in Group A without CE-boost had the lowest CNR (*p* < 0.001). There were no differences in the SNR of the portal vein in Group A without CE-Boost and Group B (*p* > 0.05).

**Conclusion:**

CE-boost can significantly improve image quality in portal vein imaging without any additional scanning settings or changes in the clinical workflow.

**Graphical Abstract:**

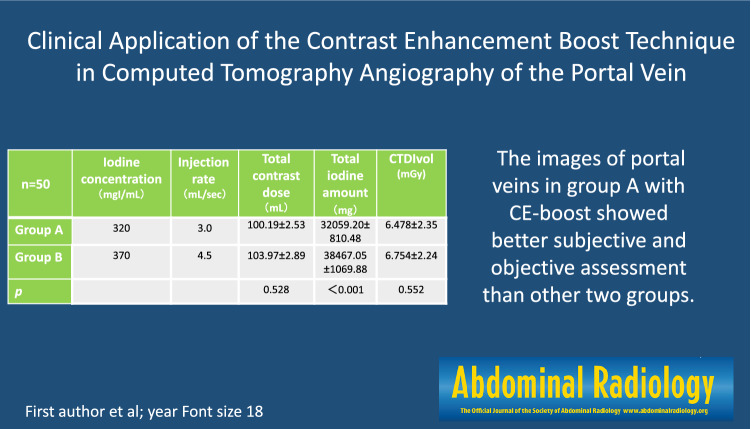

## Introduction

The portal vein supplies approximately 80% of the blood for the liver [1]. As it was the earliest established and independent of the morphology of the liver, portal vein segmentation was the most commonly used in liver anatomy and surgery [2]. Knowing the structure of the portal vein and confirming its relationship with liver tumors are important for treatment planning [3]. Computed tomography angiography (CTA), as a type of noninvasive vascular imaging technology, is widely used in imaging the vessels throughout the entire body [4, 5]. However, portal vein imaging is different from arterial imaging; it mainly relies on blood reflux, and it is difficult to reach a suitable blood concentration and thoroughly mix the contrast agent with the blood in portal veins, which would affect the quality of portal vein images [6]. In recent years, several postprocessing approaches have been developed to enhance the quality of portal vein images, such as dual-energy techniques to improve portal vein visualization via optimum contrast or lower monoenergetic keV images, which require a dedicated scanner with dual-energy equipment and postprocessing software [7–11]. Recently, a new technique named contrast-enhancement boost (CE-boost) was developed to further improve contrast enhancement on enhanced CT images based on an accurate deformable registration algorithm between noncontrast and contrast-enhanced images. An iodine image was created by removing an unenhanced CT image from a contrast-enhanced image. Then, the boost image was created by adding the iodine image to the original contrast-enhanced CT image using an automatic denoising procedure. A previous study showed that CE-boost could significantly improve the visualization of type II endoleaks after endovascular aortic aneurysm repair (EVAR) [12]. Therefore, the aim of this study was to explore the image quality improvement of the portal vein using the CE-Boost technique in abdominal-enhanced CT.

## Methods and materials

This study retrospectively investigated the portal vein images of patients who underwent routine examination for clinical reasons. Approval by the institutional ethics committee and written informed consent were waived.

### Patient population

This retrospective study included 50 patients (22 females and 28 males; age range, 18 ~ 84 years; mean age, 53.42 ± 13.21 years) in Group A who underwent routine abdominal-enhanced CT and 50 patients (22 females and 28 males; age range, 22 ~ 76 years; mean age, 52.48 ± 12.20 years) in Group B for abdominal CT angiography (CTA) scans with matched body mass index (BMI), weight, age, and sex from June to September 2021 in our hospital. The patient characteristics are listed in Table 1. The inclusion criteria were as follows: (1) age greater than 18 years, and (2) portal vein image complied with diagnosis requirements, as the subjective score was 3 or higher (Table 2). Those who had moderate to severe fatty liver disease (liver attenuation < 40 HU) and portal vein invasion were excluded [13].

**Table 1:** Patient characteristics, radiation dose, and contrast medium details

Characteristics	Group A (*n* = 50)	Group B (*n* = 50)	*t/Z*	*p*
Patient characteristic				
Age (years)	53.42 ± 13.21	52.48 ± 12.20	0.378	0.731
Gender	28 M/22F	28 M/22F		
Weight (kg)	66.79 ± 1.69	69.31 ± 1.93	−0.631	0.528
Body mass index (kg/m^2^)	24.18 ± 3.64	24.67 ± 4.17	0.422	0.673
Contrast medium parameters				
Iodine concentration (mgI/mL)	320	370		
Injection rate (mL/sec)	3.0	4.5		
Injection dose (kg/mL)	1.5	1.5		
Total contrast dose (mL)	100.19 ± 2.53	103.97 ± 2.89	−0.631	0.528
Total iodine amount (mg)	32,059.20 ± 810.48	38,467.05 ± 1069.88	−4.234	< 0.001
Radiation dose				
CTDIvol (mGy)	6.478 ± 2.35	6.754 ± 2.24	0.596	0.552

Data are expressed as mean ± standard deviation (SD). M: male; F: female; *p* < 0.05 was considered statistically significant

**Table 2:** The index and standard of subjective assessment

Score	Description
1	Too many artifacts, very high noise, only the RPV and LPV are displayed, poor opacification and unclear edge, the image of VR is blurry
2	Many artifacts, high noise, the third level of the portal veins was displayed blurry, unclear edge, the image of VR is blurry
3	Moderate artifact and noise, the distal branches of the portal vein can be seen, limited opacification and somewhat fuzzy edge, the image of VR is a little blurry
4	Minor image artifact and noise, good opacification to the segmental level with clear edge for confident diagnosis, the image of VR is a little blurry
5	No artifact, minimum image noise, excellent opacification to the segmental level with sharp edge for full confident diagnosis

### CT scan protocols

All patients were examined with the helical mode of a 320-row CT scanner (Aquilion ONE GENESIS Edition; Canon Medical Systems, Japan). All CT examinations were acquired in the craniocaudal direction with the patient in the supine position and in inspiratory breath-hold. The scan parameters were as follows: 0.5 mm × 80 rows; 0.5 s rotation time; D-field of view (FOV), L400 mm; tube voltage, 120 kVp; automatic exposure control (SURE Exposure 3D, Canon) with noise index (SD = 8 HU for nonenhanced scan and portal venous phases) was used for tube current modulation. The scanning ranged from the top of the diaphragm to the level of the anterior superior iliac spine. Patients were injected with iodinated contrast material (Group A: 320 mg I/mL, 1.5 mL/kg; Group B: 370 mg I/mL, 1.5 mL/kg) through the median cubital vein using a double-head power injector (injection rate: 3.0 mL/s in Group A and 4.5 mL/s in Group B), followed by a 30 mL saline flush at the same injection rate. In Group A, the artery phase and portal venous phase (PVP) acquisitions were performed at 28 s and 50 s, respectively, after the initiation of contrast medium administration. Intelligent tracking starting with arterial scanning was applied in Group B. A region of interest (ROI) was placed on the aorta ventralis, and the scan was started immediately when the CT value of the ROI reached the threshold of 180 HU. PVP was obtained 45.5 s after intravenous injection of contrast medium. All transverse CT images were reconstructed using adaptive iterative dose reduction 3D (AIDR 3D) with FC18 kernel for 1 mm slice thickness and 0.8 mm interval.

Radiation dose in terms of volume CT dose index (CTDIvol) of PVP in the two groups was recorded for further statistical analysis. Since the scan range might be different between abdominal-enhanced CT scans and abdominal angiography scans in clinical routine, the dose length product (DLP) and corresponding effective dose were not included for comparison.

### Image postprocessing and quality evaluation

Images in Group A were postprocessed with CE-boost (^SURE^Subtraction Iodine map, Canon Medical Systems, Japan) for further enhancement of the portal vein. Both subjective and objective assessments of the portal vein with different branches in three types of images (i.e., Group A with CE-boost and without CE-boost, Group B) were statistically analyzed.

Two experienced radiologists (8 and 10 years of abdominal diagnosis) who were unaware of the patient’s clinical information and image processing methods evaluated the image quality of the three types of images. Volume-rendered (VR), maximum intensity projection (MIP), and multiplanar reformations (MPR) were used for three-dimensional display of the vascular system. It was scored 1 to 5 according to the following four aspects: the number of segmental branches observed, the clarity of the vessel wall, and the artifact and noise level of the images [14] (Table 2).

**Fig. 1:**
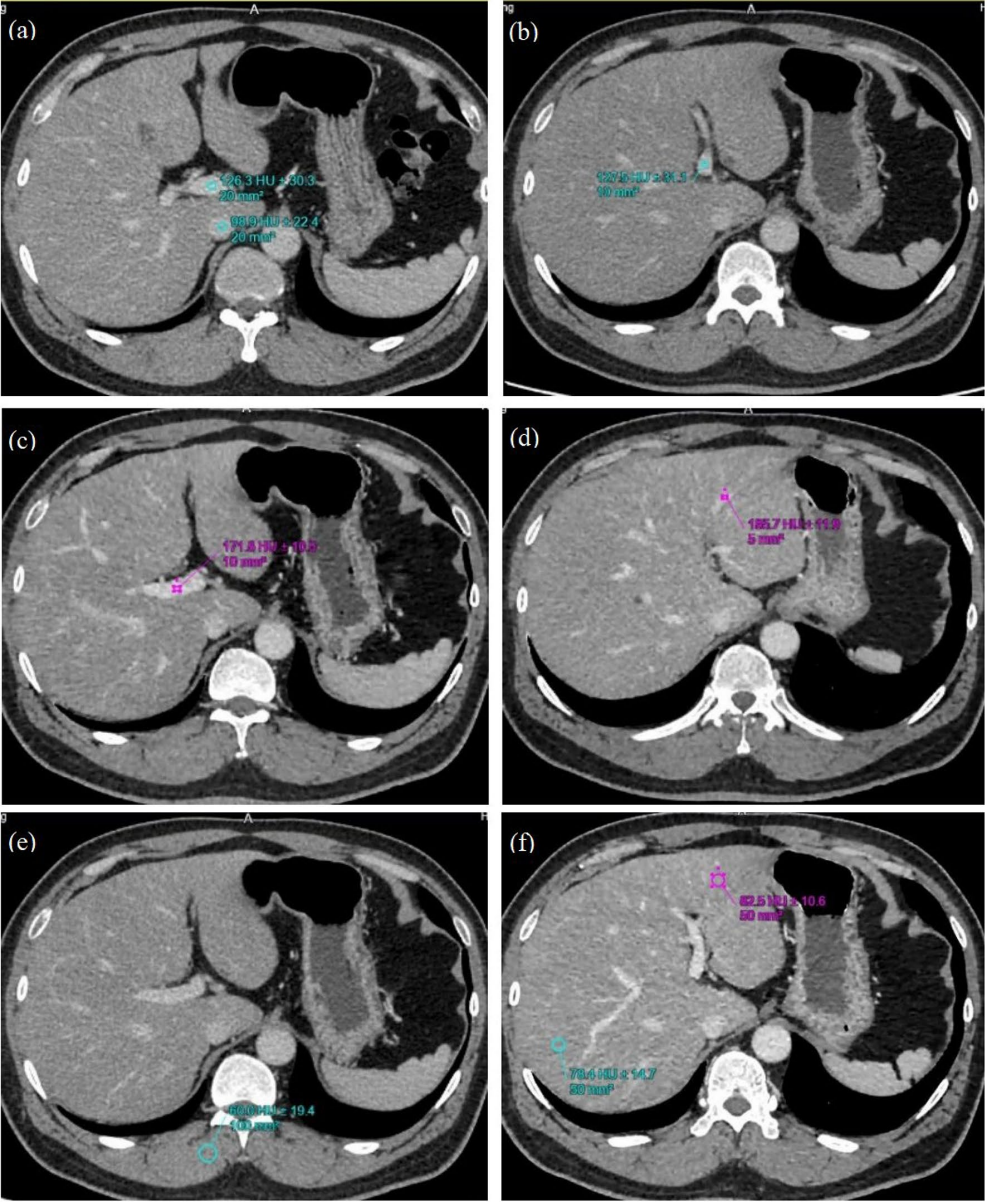
ROI location illustration: **a** liver, **b** MPV and IVC, **c** LPV, **d** RPV, **e** the third-level branch of the LPV, **f** right erector spinae

Objective evaluation included CT value, noise index (i.e., the standard deviation (SD) of the CT value), the signal-to-noise ratio (SNR), and the contrast-to-noise ratio (CNR). The SNR and CNR for vessels were calculated using the following equations: SNR = (CT_value_)/SD and CNR = (CT_vein_-CT_liver_)_/SDbackground_ [14, 15]. ROIs in the main portal vein (MPV), inferior vena cava (IVC), right portal vein (RPV), left portal vein (LPV), third-level branch of the RPV (RPV-3), and third-level branch of the LPV (LPV-3) were objectively evaluated. The ROIs were placed at the center of the veins (Fig. 1): the area of ROI in the MPV and IVC was approximately 20 mm^2^, the area of ROI in the LPV and RPV was approximately 10 mm^2^, the area of ROI in the LPV-3 and RPV-3 were approximately 5 mm^2^. The CT value and SD of the liver were calculated as the average ROI area of 50 mm^2^ of the left lobe and right lobe, and the ROI was kept away from vessels. The SD of the right erector spinae muscle (RES) with a circular ROI of 100 mm^2^ was regarded as the background SD.

### Statistical analysis

Statistical analyses were performed using R software (version 3.6.1; http://www.R-project.org). Quantitative parameters were expressed as the mean values ± standard deviations (SD). The Shapiro–Wilk test was used to assess the normality of quantitative data. In the case of normality of data, one-way analysis of variance (ANOVA) was used to analyze the difference between multiple groups. Otherwise, Kruskal–Wallis one-way ANOVA was applied. Independent samples t test with Bonferroni correction was used for multiple pairwise comparison. Otherwise, the Mann–Whitney U test with Bonferroni correction was used. The kappa value of concordance was used to measure the degree of agreement between the two experienced radiologists. Agreement was determined as follows: no agreement (kappa < 0), slight agreement (0 < kappa ≤ 0.2), moderate agreement (0.4 < kappa ≤ 0.60), substantial agreement (0.6 < kappa ≤ 0.8), and almost perfect agreement (kappa > 0.8) [12, 16]. A *p* value of < 0.05 was considered statistically significant for all tests.

## Results

### Patient characteristics

No significant difference was found between the two groups in terms of age, sex, weight or BMI (all, *p* > 0.05). The total contrast dose of the two groups had no difference (*p* = 0.528), and the total iodine amount of group B was much higher than group A (*p* < 0.001). The CTDIvol of PVP in the two groups showed no significant difference (*p* = 0.552).

### Subjective quality assessment

The subjective quality scores of the two experienced radiologists showed good consistency (kappa > 0.624, *p* < 0.001) (Table 3). The average of the two radiologists' scores was used as the final score, and then the mean and SD of the subjective score were calculated for each group of images (Group A with CE-boost: 4.68 ± 0.72; Group A without CE-boost: 4.25 ± 0.10; Group B: 4.38 ± 0.95; *p* < 0.001). The score of Group A with CE-boost was significantly higher than that of the other types of images, and there was no difference between Group A without CE-boost and Group B (*p* = 0.349).

**Table 3:** Subjective score

Group	n	Doctor1					Doctors					kappa	*p*
		1	2	3	4	5	1	2	3	4	5		
A without CE-boost	50	0	0	12	15	23	0	0	6	24	20	0.658	< 0.001
Mean^a^		4.25 ± 0.10											
A with CE-boost	50	0	0	4	10	36	0	0	0	14	36	0.624	< 0.001
Mean		4.68 ± 0.72											
B	50	0	0	7	18	25	0	0	6	18	26	0.663	< 0.001
Mean^b^		4.38 ± 0.95											
Total	150	0	0	23	43	84	0	0	12	56	82	0.664	< 0.001
*p* (compared among three groups) 0.0146													

The average of the two radiologists scores was used as the final score of each group

*p* < 0.05 was considered statistically significant

^a^represents group A without CE-boost compared to group A with CE-boost (*p* < *0.05)*

^b^represents group B compared with group A to CE-boost (*p* < 0.05)

### Objective quality assessment

**Fig. 2:**
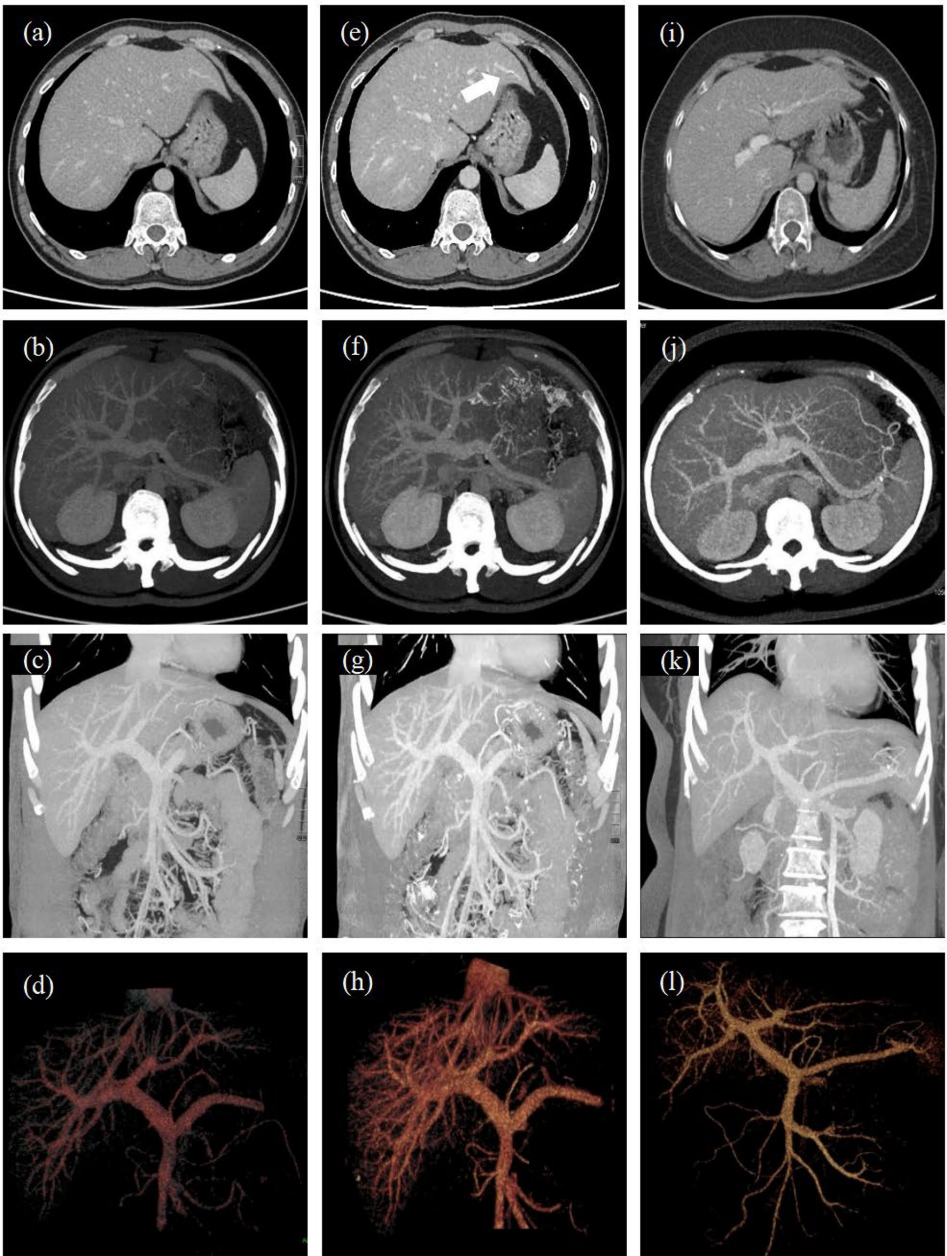
The raw and postprocessed images of the three groups (Group A without CE-boost: **a** ~ **d**, Group A with CE-boost:** e** ~ **h**, Group B: **i** ~ **l**)。The portal veins in Group A with CE-boost showed the best image quality among the three types, no matter in raw axial image or postprocessed images (i.e., MIP, VR). Group A with CE-boost showed most clearly image of the distal vessel (white arrow). MIP and VR showed more branches of portal veins in Group A with CE-boost

The results of the objective quality assessment are shown in Table 4 and Fig. 2.

**Table 4:** The objective assessment

	A without CE-boost	A with CE-boost	B	*p*
MPV				
CT value	144.69 ± 24.81^a^	200.54 ± 38.80^b^	169.72 ± 37.91^c^	< 0.001
SD	17.79 ± 4.60^a^	16.45 ± 6.57^b^	18.93 ± 2.76	0.0099
SNR	8.71 ± 2.81^a^	14.23 ± 6.54^b^	9.15 ± 2.39	< 0.001
CNR	2.75 ± 1.32^a^	5.41 ± 2.71^b^	3.64 ± 1.43^c^	< 0.001
RPV				
CT value	142.02 ± 26.01^a^	196.97 ± 39.48^b^	165.73 ± 34.46^c^	< 0.001
SD	16.92 ± 5.37^a^	15.56 ± 6.72	18.04 ± 3.42	0.0192
SNR	9.23 ± 3.39^a^	14.83 ± 5.95^b^	9.49 ± 2.69	< 0.001
CNR	2.82 ± 1.19^a^	5.34 ± 3.26^b^	3.48 ± 1.32^c^	< 0.001
LPV				
CT value	143.63 ± 23.50^a^	200.21 ± 38.66^b^	169.02 ± 34.45^c^	< 0.001
SD	15.72 ± 4.31^a^	13.87 ± 5.70^b^	17.82 ± 3.74^c^	< 0.001
SNR	9.92 ± 3.51^a^	17.39 ± 9.24^b^	9.91 ± 3.03	< 0.001
CNR	2.69 ± 1.18^a^	5.38 ± 2.61^b^	3.64 ± 1.27^c^	< 0.001
RPV-3				
CT value	145.59 ± 22.30^a^	197.52 ± 35.96^b^	164.76 ± 34.98^c^	< 0.001
SD	14.32 ± 4.92	12.99 ± 5.50	14.57 ± 3.98	0.1776
SNR	11.69 ± 5.25^a^	19.17 ± 11.41^b^	12.33 ± 4.85	< 0.001
CNR	2.81 ± 1.19^a^	5.23 ± 2.62	5.14 ± 1.90^c^	< 0.001
LPV-3				
CT value	144.18 ± 24.49^a^	195.60 ± 35.39^b^	167.07 ± 33.20^c^	< 0.001
SD	13.39 ± 4.91^a^	11.05 ± 4.81^b^	15.6 ± 4.01^c^	< 0.001
SNR	11.97 ± 4.33^a^	21.95 ± 12.51^b^	11.24 ± 3.07	< 0.001
CNR	2.76 ± 1.48^a^	5.13 ± 2.79^b^	3.54 ± 1.19^c^	< 0.001
IVC				
CT value	112.16 ± 20.07^a^	151.5 ± 28.84	138.75 ± 38.69	< 0.001
SD	19.40 ± 6.20	18.58 ± 8.89	18.50 ± 3.03	0.1142
SNR	6.35 ± 2.18^a^	9.77 ± 4.47^b^	7.71 ± 2.64^c^	< 0.001
CNR	0.89 ± 1.14^a^	2.12 ± 1.77	2.22 ± 2.01^c^	< 0.001
Liver				
CT value	96.42 ± 14.60^a^	119.12 ± 19.31^b^	90.79 ± 20.65^c^	< 0.001
SD	16.69 ± 3.14^a^	15.30 ± 4.62^b^	19.27 ± 1.37^c^	< 0.001
SNR	6.01 ± 1.56^a^	8.62 ± 3.32^b^	4.74 ± 1.46^c^	< 0.001

Data are expressed as mean ± standard deviation (SD)

*p* < 0.05 was considered statistically significant

^a^Represents group A without CE-boost compared to group A with CE-boost (*p* < *0.05)*

^b^Represents group B compared to group A with CE-boost (*p* < 0.05)

^c^Represents group A without CE-boost compared to group A with CE-boost(*p* < 0.05)

### CT value

The CT values were significantly different among the three types of images. All branches of the portal vein in Group A with CE-boost showed the highest CT value (all, *p* < 0.001), especially at the LPV (mean CT value, Group A without CE-boost: 143.63 ± 3.32 HU; Group A with CE-boost: 200.21 ± 38.66 HU; Group B: 169.02 ± 34.45). In pairwise comparisons, the CT values of the liver and all portal veins in Group A with CE-boost were much higher than those in Group A without CE-boost (all, *p* < 0.001). Compared to Group A without CE-boost, all the CT values of the corresponding vessels in Group B were higher (*p* < 0.05); there was no difference in the CT value of the liver between Group A without CE-boost and Group B (*p* = 0.31). The CT values of all comparisons except IVC (*p* = 0.072) in Group A with CE-boost was superior to those in Group B (*p* < 0.01). It was obvious that the CE-boost technique increased the CT values of the liver and vein images.

### SD

The SD of the liver showed a significant difference among the three types of images (*p* < 0.001), the lowest for Group A with CE-boost (15.30 ± 4.62 HU), followed by Group A without CE-boost (16.68 ± 3.14 HU) and highest for Group B (18.50 ± 3.02 HU), a similar trend of SD was observed for LPV (*p* < 0.001) and LPV-3 (*p* < 0.001). The SD of all branches was lower in Group A with CE-boost than in Group B (all *p* < 0.05). The SD of MPV and RPV showed no difference between Group A without CE-boost and Group B (MPV, *p* = 0.567; RPV, *p* = 0.609). SD of MPV in Group A with CE-boost (16.45 ± 6.57 HU) was lower than that in Group B (18.93 ± 2.76 HU, *p* = 0.0165); however, the SD of RPV showed no difference between Group A with CE-boost and Group B (*p* = 0.052). In summary, Group A with CE-boost had significantly reduced image noise in the portal vein.

### SNR

For the analysis of SNR, significant differences were observed among the three types of images (all, *p* < 0.001). Group A with CE-boost had a much higher SNR than the other two types of images, especially at LPV (17.39 ± 9.24) and LPV-3 (21.94 ± 12.51). In pairwise comparisons, there were no differences with SNR in MPV or branches of portal vein between Group A without CE-boost and Group B (all, *p* > 0.05). The SNR of the liver in Group B (4.74 ± 1.15) was inferior to that in Group A without CE-boost (6.00 ± 1.56) (*p* < 0.001). The SNR of IVC with Group A without CE-boost (6.35 ± 2.18) was higher than that with Group B (4.74 ± 1.46) (*p* = 0.041). Group A with CE-boost had the highest SNR, and Group A without CE-boost had no difference in SNR compared with SNR in Group B for portal veins.

### CNR

There were significant differences in CNR among the three types of images (all, *p* < 0.001). Group A with CE-boost had the highest CNR of MPV and most branches, especially in the LPV (7.62 ± 1.61), which was nearly twice that in Group B (3.63 ± 1.27) and Group A without CE-boost. There were no differences in the CNR of the IVC and the RPV-3 between Group A with CE-boost and Group B (both, *p* = 1). Comparing Group A without CE-boost with Group B, the CNR of all comparisons in Group B was higher (*p* < 0.05). CE-boost could significantly improve the CNR of the portal vein images.

## Discussion

Our study demonstrated that CE-boost could significantly increase the CT value and decrease image noise to obtain a higher SNR and CNR for the portal vein in a routine abdomen-enhanced scan with a lower concentration of iodine contrast medium.

The quality of the portal vein image depends on the enhancement degree of the portal vein and the contrast between the portal vein and its surrounding liver parenchyma. It is common to improve the contrast ratio of the portal vein by increasing the amount and iodine concentration of the contrast agent when doctors need to observe the portal vein and its relationship with adjacent lesions in clinical work [17], similar to the scan protocol of Group B in this study. The comparison of Group A without CE-boost and Group B illustrated this, and most branches of the portal vein in Group B appeared brighter than those in Group A without CE-boost. However, we found that image noise of portal veins in Group B was higher than Group A without CE-boost; therefore, although the CT value of Group B was higher than that of Group A without CE-boost, there was no difference in SNR between Group A without CE-boost and Group B. Studies [18, 19] have shown that a high risk of kidney damage is associated with high concentrations of contrast agents. Increasing the contrast agent concentration and total dose is not the preferred method to improve the image quality of the portal vein. Many efforts have been made to reduce the amount of contrast media as much as possible while maintaining portal vein image quality [17, 20]. Han et al. [21] used lower monoenergetic images combined with adaptive statistical iterative reconstruction (ASiR) in dual-energy spectral CT to reduce the iodine amount in CT portal venography (CTPV). They found that 50 keV images with ASiR reconstruction could reduce the total iodine amount by 52% while maintaining good image quality. Miyoshi et al. [8] found that low tube voltage (70 kVp) in combination with a half-dose iodine load using a low-concentration contrast agent and an iterative reconstruction algorithm in high tube output dual-source CT may improve the contrast enhancement and image quality in multiphasic dynamic CT of the abdomen in patients weighing less than 71 kg. Yoon et al. [22] investigated lesion detection capability and image quality between the standard-dose group and the double low-dose group (i.e., 30% reductions in both radiation and contrast media), they found that 50 kiloelectronvolt images of the double low-dose group showed better results.

CE-boost is a new technique to further increase contrast enhancement on enhanced CT images based on an accurate deformable registration algorithm for noncontrast and contrast-enhanced images. The CE-boost technique solved the problem that the same layer of the silhouette cannot be prepared for matching in nonenhanced and enhanced scanning caused by the patient's autonomic or physiological movement. In this study, both subjective scores and objective values showed that Group A with CE-boost had better image quality than that without CE-boost. We hypothesized that if the images in the scan protocol of Group A could obtain better portal vein images with CE-Boost postprocessing than those in Group B, then radiologists could observe the lesions in the portal vein and liver parenchyma simultaneously in PVP of routine abdominal enhancement scans. The subjective indices of most vessels were superior in Group A with CE-boost when compared to Group B. The CNR of RPV-3 showed no difference between Group A with CE-boost and Group B. We inferred that the CT value of the distal veins was low and could not show much enhancement even with CE-boost postprocessing. The CT value and CNR of the IVC were not different between the two types. The reason might be that the time of hepatic vein imaging is later than that of the portal vein imaging, and the hepatic vein might not be fully filled at the time of the two scan protocols mentioned above. Therefore, routine abdominal-enhanced CT with the postprocessing technique of CE-boost could improve the CT value, SNR, and CNR of most portal veins and reduces the SD of portal veins; it would provide a better image of portal veins than abdominal computed tomography angiography, which had a higher injection rate and iodine concentration.

There were several limitations in this study. First, we only matched the BMI of the two groups but did not use a large sample grouped by BMI, as the X-ray penetration ability was weakened with increasing BMI [23]; moreover, there was an inverse relationship between the degree of enhancement and body habitus. For the next step, a larger population with a wider range of BMI distributions would be recruited for further study. Second, there was no personalized design for scan delay time and contrast agent injection rate, which would obtain the optimized image quality. Third, the diagnostic performance of liver diseases was not evaluated; therefore, liver lesion visualization and diagnosis will be further deeply assessed with the CE-boost technique.

## Conclusion

CE-boost could significantly improve image quality in CTPV without any additional scanning settings or changes in the clinical workflow to obtain comparable or even better visualization of the portal vein.
